# Search for Dispersed Repeats in Bacterial Genomes Using an Iterative Procedure

**DOI:** 10.3390/ijms241310964

**Published:** 2023-06-30

**Authors:** Eugene Korotkov, Yulia Suvorova, Dimitry Kostenko, Maria Korotkova

**Affiliations:** 1 Institute of Bioengineering, Research Center of Biotechnology of the Russian Academy of Sciences, Bld. 2, 33 Leninsky Ave., 119071 Moscow, Russia; suvorovay@gmail.com (Y.S.); dk0stenko@yandex.ru (D.K.); 2 Moscow Engineering Physics Institute, National Research Nuclear University MEPhI, 31 Kashirskoye Shosse, 115409 Moscow, Russia; bioinf@rambler.ru

**Keywords:** dispersed repeats, bacteria, genome, dynamic programming, iteration

## Abstract

We have developed a de novo method for the identification of dispersed repeats based on the use of random position-weight matrices (PWMs) and an iterative procedure (IP). The created algorithm (IP method) allows detection of dispersed repeats for which the average number of substitutions between any two repeats per nucleotide (*x*) is less than or equal to 1.5. We have shown that all previously developed methods and algorithms (RED, RECON, and some others) can only find dispersed repeats for x ≤ 1.0. We applied the IP method to find dispersed repeats in the genomes of *E. coli* and nine other bacterial species. We identify three families of approximately 1.09 × 10^6^, 0.64 × 10^6^, and 0.58 × 10^6^ DNA bases, respectively, constituting almost 50% of the complete *E. coli* genome. The length of the repeats is in the range of 400 to 600 bp. Other analyzed bacterial genomes contain one to three families of dispersed repeats with a total number of 10^3^ to 6 × 10^3^ copies. The existence of such highly divergent repeats could be associated with the presence of a single-type triplet periodicity in various genes or with the packing of bacterial DNA into a nucleoid.

## 1. Introduction

Dispersed repetitive DNA sequences constitute a significant portion of existing genomes. In the human genome, more than one third is occupied by dispersed repeats, which are primarily copies of transposable elements [1], whereas in other organisms, the proportion could be much higher [2,3]. Accurate localization of dispersed repeats in the sequenced genome can help determine the functional significance and evolutionary origin of genomic sequences. Considering that the genomes of many eukaryotic organisms have already been sequenced and those of all living organisms are expected to be sequenced in the next decade according to the Earth BioGenome Project [4], the search for dispersed repeats in various genomes emerges as an important task of bioinformatics.

At present, many mathematical methods and algorithms have been developed for the identification of dispersed repeats [5]. Most of the programs contain two independent parts: the first (A) generates a dispersed repeat sequence, whereas the second (B) searches the genome under study for sequences similar to those generated in part A. Part A can be of two types: A1 uses a library of already published dispersed repeats, whereas A2 uses dispersed repeats created de novo by various mathematical methods. The dispersed repeats of the library or those created de novo are used in part B to search for similar sequences in the genome.

Programs such as RepeatMasker [6], Censor [7], and MaskerAid [8] are A1-based. The mathematical methods implemented in these programs detect repeats in the genomes by comparison with Repbase [9], a database of repetitive sequences. Thus, the detection of new repeats by these methods is limited to the ones existing in the library.

Programs such as RED [10], Recon [11], PILER [12], RepeatScout [13], and RepeatFinder with REPUter [14] are A2 based (reviewed in [5]). These methods create de novo sequences of dispersed repeats using similarity search, word counting, and signature-based approaches. The effectiveness of the A2-based methods in the discovery of new families of dispersed repeats and/or analysis of new genomes is superior to that of A1-based methods.

Many similar search programs such as BLAST [15], FASTA [16], MEGA [17], and nHMMER [18] are based on part B. The use of nHMMER allows the search for dispersed repeats by multiple alignment, which seems to be preferable to searches organized by consensus sequence or individual repeats. COFFEE [19] or MUSCLE [20] can be used to construct multiple alignments for nHMMER as these methods allow calculation of statistically significant multiple alignments with up to *x* < 2.4, where *x* is the average number of substitutions per nucleotide when comparing dispersed repeats of the same family [21]. However, for this purpose it is better to use MAHDS [21] which allows for the discovery of a statistically significant multiple alignment with *x* < 4.4, thus providing detection of more divergent copies of dispersed repeat families.

According to functional significance and structural features, all existing dispersed repeats identified in eukaryotic genomes can be roughly divided into five classes: short interspersed nuclear elements (SINEs), long interspersed nuclear elements (LINEs), long terminal repeats (LTRs), DNA transposons, and others [1]. Prokaryotic genomes, which are composed mainly of coding sequences, contain significantly fewer dispersed repeats. Thus, in the *E. coli* genome the average distance between genes is about 118 nucleotides [22] and the proportion of dispersed repeats is only 0.7%. Palindromic sequences with a length of 40 bp (called REP, BIME, or PU) represent the largest class of repeats in the *E. coli* genome. The longest repeated sequences in *E. coli* K-12 are five Rhs elements ranging from 5.7 to 9.6 kb [22]. The *E. coli* K-12 genome also contains small transposable elements called insertion sequences (ISs). However, dispersed repeats with a length of more than 300 bases and a large number of copies have not been detected in the *E. coli* genome.

An important question arises as to whether it is possible to identify all dispersed repeats existing in eukaryotic or prokaryotic genomes. The problem is that repeats can accumulate a large number of mutations, such as base substitutions or insertions and deletions (indels). As a result, the similarity between repeats from the same family can become extremely weak and such repeats can be missed. Therefore, effective methods to identify highly divergent repeats in the genome should be developed.

The search for dispersed repeats can be affected by the limitations of the methods based on part A. The performance of the A1-based methods obviously depends on the volume of created repeat libraries, which in turn is determined by the data obtained by experimental or theoretical studies, while the effectiveness of the A2-based methods is limited by the possibility to find dispersed repeats de novo. Let us consider a simple example of searching for any two repeats of length *L*_1_ in a sequence of length *L*. To perform this task, we select two windows of length *L*_1_ in the sequence of length *L* and compare their sequences. The first window runs from the beginning to the end of sequence *L*, and the second runs from the end of the first sequence to the end of sequence *L*. As a result, the number of comparisons between the two sequences (*N*) is proportional to *L*^2^/2. In this case, the probability of detecting random similarity between two sequences of length *L*_1_ (*p*) should be no more than *k*/*N* ≈ 0.01. To exclude noise in the form of random pairwise similarities, we take *k* = 0.01, which means that the probability of finding random similarity is 1%. From probability *p*, we can estimate the level of statistically significant similarity. Let us first calculate the argument of normal distribution *Z*_0_ for which *P*(*Z* > Z_0_) = *p*. Based on *Z*_0_, we can estimate the number of identical bases *s*_0_ for which the similarity of two sequences of length *L*_1_ is considered statistically insignificant (or noise): $s_{0}=\bar{s}+z_{0}\sqrt{L_{1}t(1-t)}$, where *t* is the probability of random similarity between two nucleotides taken as 0.25. In the case of a bacterial genome, *L* = 4 × 10^6^. If repeat length *L*_1_ is 300, then probability *p* is approximately 10^−15^, which corresponds to *Z*_0_ ≈ 8.0; thus, *s*_0_ = 135, which corresponds to 45% similarity and *x* ~ 0.55 (formula 11 in [23]). This means that if a family of dispersed repeats has accumulated many mutations and their similarity is less than 45%, it is very difficult to detect such a family de novo in a 4 × 10^6^-base long sequence by A1 methods. A similar *x* value is shown in [5]. It should be noted that, in reality, *x* may be smaller because in addition to base substitutions, indels could be present in dispersed repeats. The use of word counting and signature-based methodologies to search for dispersed repeats cannot significantly improve the situation, because at *x* = 0.75 sequence similarity is already at the level of random noise (~25%) and word frequencies may differ insignificantly from those expected for random sequences.

In this study, we showed that the known de novo methods could find dispersed repeats with *x* ≤ 1.00, whereas part B-based methods such as nHMMER could do the same with *x* ≤ 3.0 using a previously created multiple alignment, indicating that the main constraints on the identification of dispersed repeats are related to part A. Thus, a family of unknown dispersed repeats in the genome which has accumulated a large number of base substitutions and indels (*x* > 1.0) is unlikely to be detected by A2-based methods. Consequently, the application of part B-based methods is impossible because there is no sequence, consensus, or multiple alignment to search for the dispersed repeats of such a family in the genome. Therefore, highly divergent repeat families (*x* > 1.0), which could be present in already sequenced genomes, cannot be detected because of the limitations of A2-based methods.

Here, we report a method for identifying dispersed repeats based on the use of random positional weight matrices (PWMs) and an iterative procedure (IP). The developed algorithm allows for the detection of dispersed repeats with *x* ≤ 1.5, which significantly exceeds the capacity of all modern methods based on part A2 (*x* ≤ 1.0). We applied the new IP method to search for dispersed repeats in the genome of *E. coli* and nine other bacterial species and showed that the bacterial genomes contained families of dispersed repeats with copy numbers >10^3^ and lengths of 400–600 bases.

We chose bacterial genomes for this analysis because they lack long dispersed repeats (>300 b.p.) with copy numbers greater than 10^3^ and therefore the results obtained cannot be related to known families of dispersed repeats. Additionally, bacterial genomes are significantly shorter than eukaryotic genomes and their analysis does not require very high computational power. At the same time, weakly similar dispersed repeats in bacterial repeats may be present due to the stacking of bacterial DNA in the nucleoid [24]. Possible functional significance and the origin of the dispersed repeats are discussed.

## 2. Results

### 2.1. Using Model Sequences for the IP Method

We generated a random sequence *S_test_* with a length of 4 × 10^6^ bases and randomly inserted it into sequences from set *Q*(*x*), which contained 10^3^ sequences with *x* substitutions relative to each other. Overall, 500 sequences from set *Q*(*x*) were introduced into sequence *S_test_* in the forward orientation and 500 in the reverse orientation. Then, the IP method was applied to identify the repeats. The results shown in Table 1 (where columns 1–4 show the number of repeats found in the first four families) indicate that the repeats were detected in the first and second families (columns 1 and 2), whereas the other two families (columns 3 and 4) contained sequences combined randomly. The level of random noise was 145 ± 35 sequences in all four generated repeat families. The results in Table 1 indicate that IP clearly separated forward and reverse sequences into two families and consistently detected two repeat families with *x* up to 1.5.

The search for sequences such as the PWM (Section 4.3) was performed in only one direction. Therefore, forward and reverse repeats could create two separate families; as a result, to conclusively determine the number of the identified families, we needed to check for possible association of the two PWM families into a single family by taking into account inversion and complementarity. Such analysis indicated that the similarity of the PWMs of the three dispersed repeat families was statistically insignificant, indicating that the identified families cannot be combined. Below (Section 2.3), we show that the reason for this is the association of the families of dispersed repeat families found in the *E. coli* genome with triplet periodicity, which in different families is not similar with regard to inversion and complementarity.

### 2.2. Comparison of the IP Method with RED, RECON, RepeatMasker, BLAST, and nHMMER

**Figure 1:**
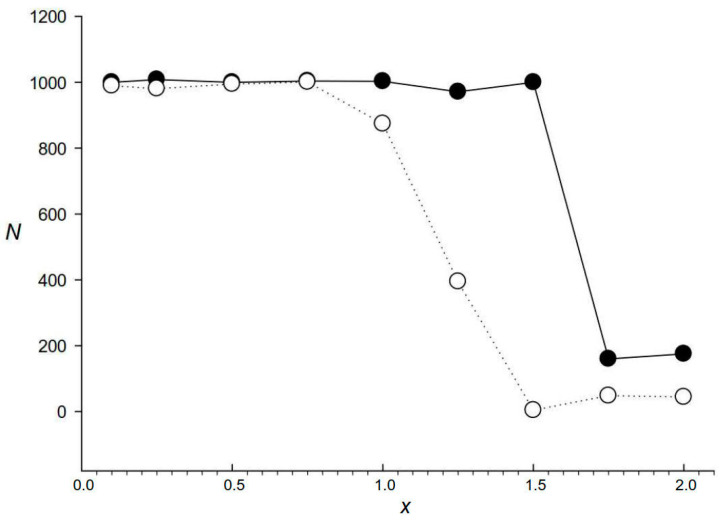
Comparative performance of the IP and RED methods in search for dispersed repeats in artificial sequences. The search was performed in an artificial sequence of 4 × 10^6^ bases containing 10^3^ repeats (each of 600 bases but with different *x*), which were randomly inserted from set *Q*(*x*) containing repeats with the same number of random mutations relative to original sequence *S*_m_ as well as indels at random positions. Black and white circles indicate the IP and RED methods, respectively; *x* is the average number of substitutions per nucleotide and *N* is the number of repeats identified.

A previous study has indicated that the known methods of de novo search for dispersed repeats cannot detect them if the number of mutations in the dispersed repeats that originate from a common ancestor exceeds 25–30 [5], which corresponds to *x* ≤ 0.6. Our calculations (Section 1) showed that by using pairwise comparison, such a search is possible at *x* ≤ 0.55, which roughly corresponds to the result obtained in [5]. We chose RED (which uses K-mers) [10] as one of the popular algorithms for finding repeats and applied it to identify 10^3^ artificial repeats of 600 bases randomly scattered across a sequence of 4 × 10^6^ bases. A total of nine sequences were created, for which dispersed repeats had different numbers of mutations relative to each other, i.e., *x* = 0–2.0. Figure 1 shows that RED found 100%, 40%, and practically 0% of dispersed repeats with *x* ≤ 1.0, *x* = 1.25, and *x* ≥ 1.5, respectively, indicating that RED could reliably identify dispersed repeats only with *x* ≤ 1.0. At the same time, the application of the IP method to the nine artificial sequences resulted in the detection of dispersed repeats with *x* ≤ 1.5 (Figure 1), which is a better result than that of RED.

We also examined the performance of RECON [11] in finding families of dispersed repeats with different degrees of divergence. The results indicate that, similar to RED, RECON could find dispersed repeats only with *x* ≤ 1.0. However, at *x* = 1.00, RECON, instead of finding just one repeat family, detected 108 families containing an overall number of 825 repeats, which is an incorrect result. Therefore, RECON could detect one family of repeats only with *x* ≤ 0.8.

In a previously study, the same analysis was performed using the RepeatMasker program, and the results indicate that it finds almost 100% of dispersed repeats for *x* ≤ 0.5 but 25% and less than 5% for *x* = 0.75 and for *x* = 1.0 [25].

Many of the de novo methods mentioned in [5] use the BLAST program to compare sequences with themselves and then perform the assembly of dispersed repeats from the found similarities. Therefore, it was interesting to analyze the capability of BLAST to search for dispersed repeats in the same artificial sequence *S*(*x*) (4 × 10^6^ bases) carrying 10^3^ randomly inserted 600 base repeats from set *Q*(*x*), which had the same number of random mutations relative to original sequence *S_m_*, two indels at random positions, and *x* substitutions per nucleotide between repeats. The word_size parameter in BLAST was chosen to be 4, which gave the best result. To evaluate the effectiveness of BLAST, we randomly created 100 sequences of *S*(*x*) containing repeats from different *Q*(*x*) (E_value was chosen to be 100). E-value is the number of expected hits of similar score that could be found just by chance. Table 2 shows the numbers of average repeats per sequence *S*(*x*) for each *x*. The results indicate that BLAST could find pairwise similarity for *x* ≤ 1.0 but failed to do so for *x* > 1.0.

Thus, previous findings with the de novo A2-based methods [5] and the results of the present analysis suggest that the currently used methods can identify dispersed repeats with *x* ≤ 1.0 but skip those with *x* > 1.0. In contrast, the IP method can identify repeats with *x* ≤ 1.5, i.e., those missed by the other methods.

We also compared the performance of the IP method with that of nHMMER, which is one of the best part B-based methods to find dispersed repeats with already known multiple alignments. In this test, we aligned all 10^3^ sequences from set *Q*(*x*), and since the placement of indels in these sequences with respect to *S_m_* was known, it was not difficult to construct a multiple alignment, which was used by nHMMER to create a hidden Markov model and search for dispersed repeats in sequence *S*(*x*).

**Figure 2:**
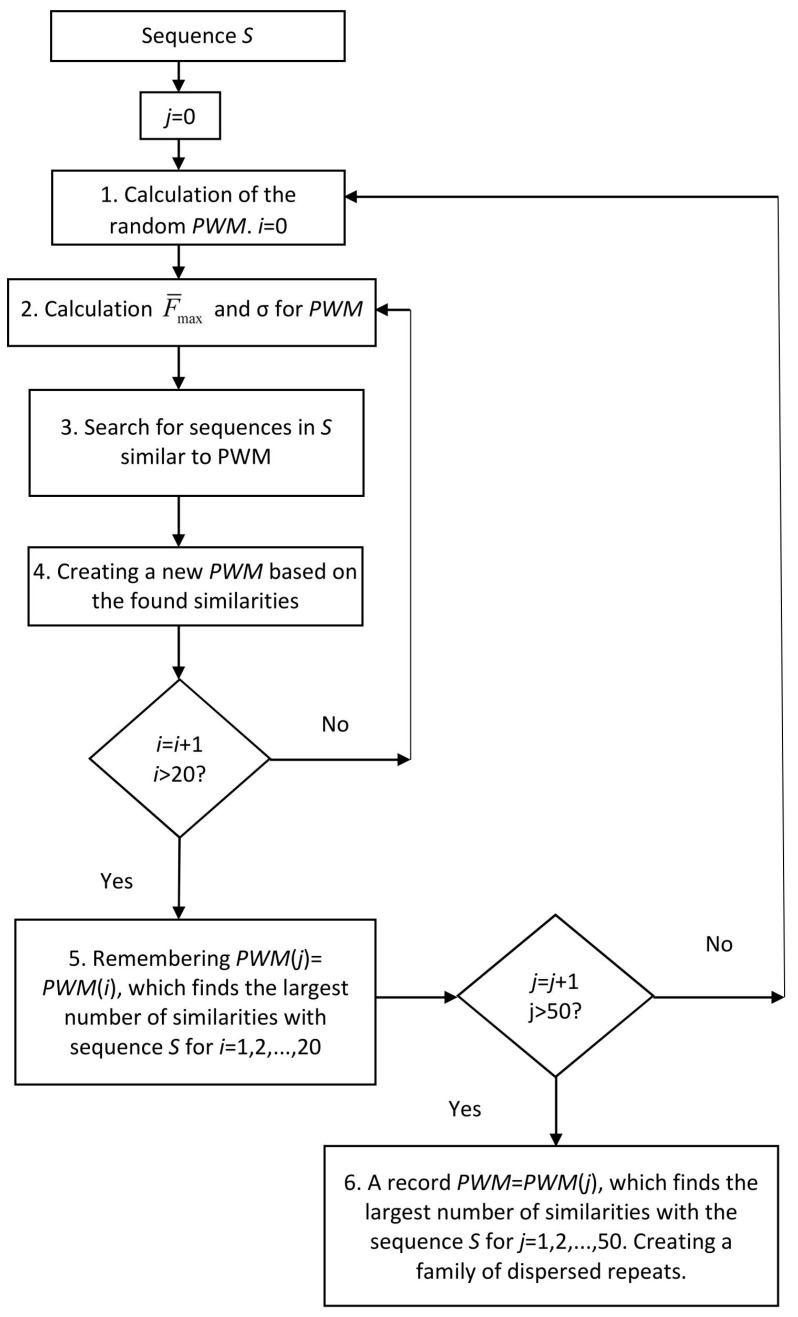
Diagram of the IP algorithm used in this study to search for de novo dispersed repeat.

We also searched for dispersed repeats in sequence *S*(*x*) using the IP method. To correctly compare the IP method with nHMMER in search for repeats with known multiple alignments, we used *S_m_* as a sequence from the library to create a PWM (Figure 2, step 4), when indices *i* and *j* were limited to 1 (i.e., one cycle of dispersed repeat search).

The created PWM had a length of 600 bases and 16 rows and was filled in as: *PWM*(*n*,*i*) = *PWM*(*n*,i) + 1 for all *i* from 2 to 600 (here, *n* = *s_m_*(*i*−1) + 4*s_m_*(*i*) and *i* is the column number). Then, for *i* = 1 *n* = *s_m_*(600) + 4*s_m_*(1).

The results shown in Table 2 indicate that the IP method, similar to nHMMER, could find dispersed repeats using a known alignment. Both methods perform reliably up to *x* ≤ 3.0; however, the IP method was slightly more efficient at *x* = 3.0.

### 2.3. Search for Dispersed Repeats in the E. coli Genome Using the IP Method

**Figure 3:**
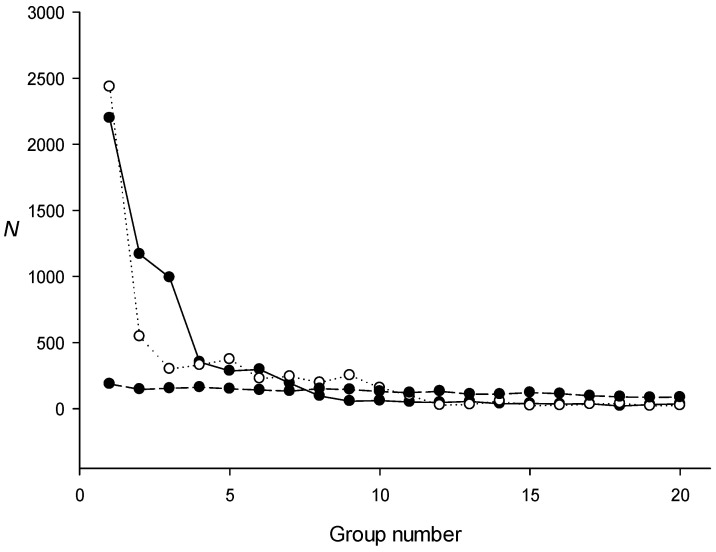
The number of repeats in the groups created for the *E. coli* genome. Black circles on a continuous curve indicate the groups for the escherichia_coli_str_k_12_substr_mg1655_gca_000005845 genome; white circles indicate the size of the groups created for the same genome, in which the codons in coding sequences are mixed randomly; black circles on a discontinuous curve indicate the size of the groups created for a random sequence of the same length as the escherichia_coli_str_k_12_substr_mg1655_gca_000005845 genome. *N* is the number of repeats identified.

The escherichia_coli_str_k_12sbstr_mg1655_gca_000005845.ASM584v2.49 sequence was obtained from http://bacteria.ensembl.org/index.html/, accessed on 1 January 2023. To search for dispersed repeats in the *E. coli* genome with the IP method, we used length *L*_1_ = 600 bases (Section 4.1). The results shown in Figure 3 indicate that, for a random sequence, the IP method generated families of 145 ± 35 repeats, because the iterative algorithm (Figure 2) can always capture a certain number of sequences and build PWMs. In the case of the *E. coli* genome, the respective numbers of dispersed repeats found in the three families were 2239, 1170, and 1024. The volume of the other repeat families was close to random, and the probability of finding families of such volume in a random sequence of the same length as the genome is extremely low. The coordinates of the found repeat families and their alignment with the PWM (sequence *S*_2_, Section 4.3 and Section 4.5) are shown in Supplementary Materials in additional files fam1.txt, fam2.txt, and fam3.txt, and the PWMs created for these families are shown in files pwm1.txt, pwm2.txt, and pwm3.txt.

We also constructed an artificial sequence based on the *E. coli* genome, where the codons in each gene were randomly shuffled to ensure that any similarity of the coding sequences was absent but where the triplet periodicity was preserved because the first, second, and third codon positions did not change [26]. In Figure 3, the volume of the created families for this sequence is indicated by white circles. The results show that if the codons in the genes were conserved, dispersed repeat families of sufficiently large volumes could still be created, indicating that the identified repeat families were associated with the triplet periodicity of coding sequences [27].

**Figure 4:**
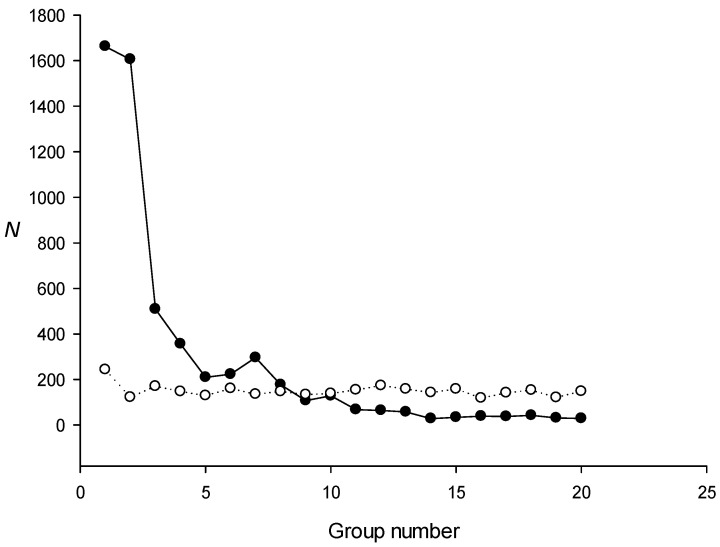
The number of repeats in the coding sequences of the *E. coli* genome. Black circles indicate groups created for the escherichia_coli_str_k_12_substr_mg1655_gca_000005845 genome, in which all non-coding sequences were randomly mixed; white circles indicate the size of the groups created for the randomly mixed genome. *N* is the number of repeats identified.

Next, we created an artificial sequence containing the *E. coli* genome in which all non-coding sequences were randomly mixed and used it to search for dispersed repeat families with the IP method. Figure 4 shows that, in this case, we could still find families of dispersed repeats, indicating that the non-coding regions in the *E. coli* genome do not contain a significant number of dispersed repeats. This result was verified by calculating the proportion of non-coding regions in dispersed repeats of families 1, 2, and 3 (Table 3), which confirmed that most of the found repeat families were not associated with non-coding sequences.

**Figure 5:**
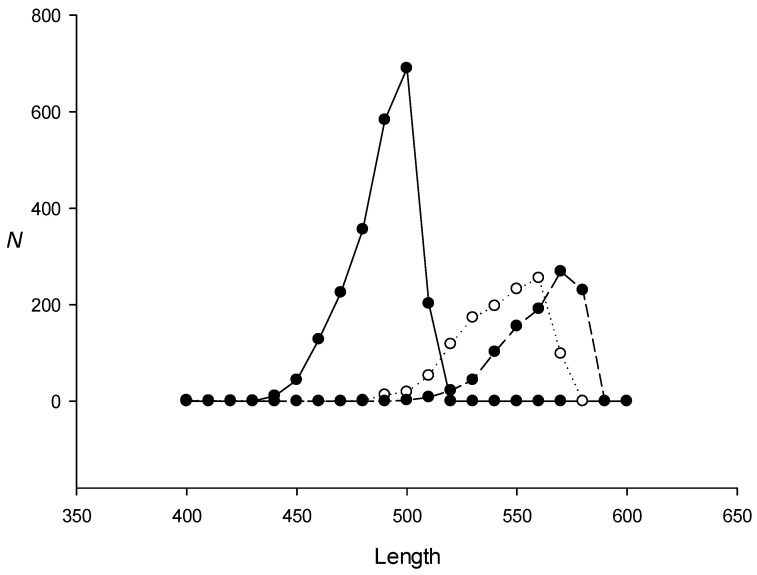
Length distribution of dispersed repeats found in the escherichia_coli_str_k_12_substr_mg1655_gca_000005845 genome. Repeats of families 1, 2, and 3 are indicated by black circles on a continuous line, white circles, and black circles on a discontinuous line, respectively.

Next, we analyzed the length distribution of the repeats in each of the three families (Figure 5). The repeats of the first family (485 bases) were slightly shorter than those of the second and third families (548 and 564 bases, respectively).

### 2.4. Triplet Periodicity of Dispersed Repeat Families in the E. coli Genome

Next, we investigated the triplet periodicity in the sequences of the three found repeat families. For this, we filled in matrix *M*(3,4) for each repeat sequence: *s*_1_(*i*)) = *M*(*f*(*s*_2_(*i*)) + 1, *s*_1_(*i*)) + 1 for *i* from 1 to *L*, where *L* is the repeat length, *s*_1_(*i*) is a sequence element of the found repeat (sequence *S*_1_, Section 4.3), and *s*_2_(*i*) is a column sequence element of the PWM (sequence *S*_2_, Section 4.3). We calculated function *f*(*s*_2_(*i*)) = *s*_2_(*i*)−3int((*s*_2_(*i*)−0.1)/3.0) and found that it was 1, 2, or 3. If *s*_1_(*i*) or *s*_2_(*i*) were equal to zero (deletion), then 1 was not added to *M*(*f*(*s*2(*i*)) but added to *i*. After determining matrix *M*, we calculated mutual information *I* as:

(1) $$I=\sum_{i=1}^{3}\sum_{j=1}^{4}m(i,j)\ln m(i,j)-\sum_{i=1}^{3}X(i)\ln X(i)-\sum_{j=1}^{4}Y(j)\ln Y(j)+L\ln L$$

where *m*(*i*,*j*) is an element of matrix *M*(3,4), $X(i)=\sum_{j=1}^{4}m(i,j)$, $Y(j)=\sum_{i=1}^{3}m(i,j)$, and $L=\sum_{i=1}^{3}\sum_{j=1}^{4}m(i,j)$.

**Figure 6:**
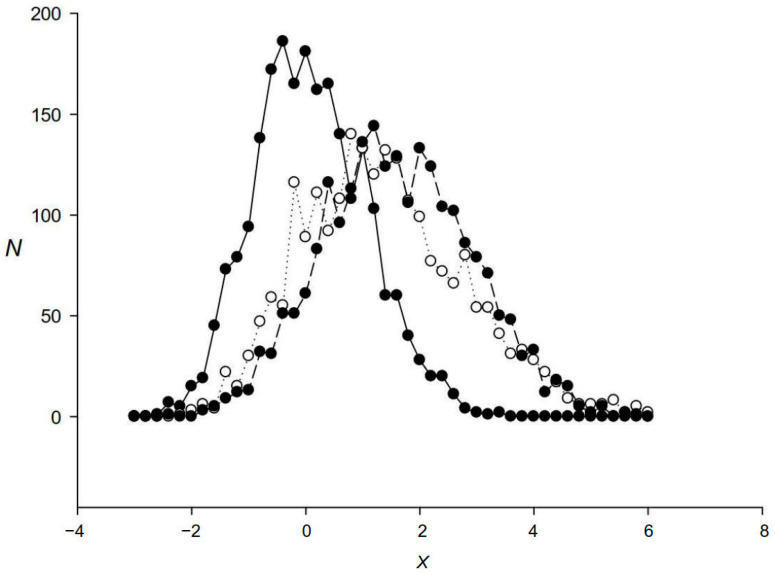
Distribution of the dispersed repeats from the first group according to the level of triplet periodicity. Here, *X* is the argument of normal distribution indicating the level of statistical significance of triplet periodicity. Black circles on a continuous line show triplet periodicity calculated for randomly mixed sequences from the first group; white circles show triplet periodicity for the first group sequences found in the escherichia_coli_str_k_12_substr_mg1655_gca_000005845 genome without alignment with the PWM for this family (sequences without indels as they are in the genome); black circles on a dashed line show the level of triplet periodicity in the sequences that are a part of the alignments with the first group PWM (sequences with indels).

Then, we calculated the argument of normal distribution *Z* = (4*I*)^0.5^ − (11.0)^0.5^ for each sequence in the repeat family and plotted *Z* distribution for all family members. Figure 6 shows the distribution for the first repeat family. For randomly shuffled sequences, the *Z* distribution was close to normal (black circles), but for repeat sequences without alignment with the PWM, the distribution was markedly shifted to the right (white circles), indicating that the coding sequences had triplet periodicity [26]. The *Z* distribution for sequences aligned with the PWM was shifted even more to the right compared with the two distributions mentioned above (black circles on a dashed line; Figure 6), indicating that the alignment of repeats with the PWM results in a clearer triplet periodicity.

We combined all matrices *M* from all sequences for each family into one matrix, created matrices *M*_1_, *M*_2_, and *M*_3_, and converted each matrix element into a normal distribution argument using normal approximation for binomial distribution. For this, we calculated partial sums of *X*(*i*) and *Y*(*j*) as we did in Figure 6 and determined probabilities *p*(*i*,*j*) = *X*(*i*)*Y*(*j*)/*L*_2_ and the argument of normal distribution *z_k_*(*i*,*j*) = {*m_k_*(*i*,*j*)-*Lp*(*i*,*j*)}/{*Lp*(*i*,*j*)(1.0-*p*(*i*,*j*)}^0.5^ (where *k* indicates the number of the dispersed repeat family). The resulting matrices are shown in Table 4; column numbers are *s*_2_(*i*)mod(3), where *s*_2_(*i*) is the column sequence element of the PWM (sequence *S*_2_, Section 4.2). It should be noted that the column numbers in matrices *M*_1_, *M*_2_, and *M*_3_ are unrelated to the reading frame in the genes because there are indels in sequences *S*_1_ and *S*_2_. From the matrices, we could conclude that the nucleotides were distributed extremely unevenly across the matrix positions. Thus, the first group of dispersed repeats contained more than expected nucleotides C and G in the first position, C in the second position, and A and T in the third position.

### 2.5. Comparison with Nucleoid-Associated Protein-Binding Sites

It is known that the spatial structure of bacterial genomes, including that of *E. coli*, is maintained by so-called nucleoid-associated proteins (NAPs). By binding to DNA, these proteins help stabilize and compact DNA and can also play regulatory functions. Several such proteins, with different properties and DNA specificity, are known currently [24]. Experimental binding maps have been constructed for some NAPs using chip-seq methods. It is possible that the DNA regions with remote similarity identified in this study may be the binding sites for various NAPs. To test this hypothesis, we compared the intervals found here with the binding sites of some NAPs (such as Fis, H-NS, and Ihf) whose binding site coordinates were obtained from [28,29]. The intersection of these coordinates was determined using the bedtools program [30]. The results reveal that there was no statistical difference between the numbers of intersections of NAP-binding sites with the found repeat families and with randomly located dispersed repeats of these families, indicating the absence of a statistically significant intersection of the found intervals with NAP-binding sites.

### 2.6. Search for the Families of Dispersed Repeats in the Genomes of Other Bacteria

To confirm that dispersed repeat families exist not only in *E. coli* but also in other bacteria, we applied the IP method to search for dispersed repeats in the genomes of the following bacterial species: *Azotobacter vinelandii*, *Bacillus subtilis*, *Clostridium tetani*, *Methylococcus capsulatus*, *Mycobacterium tuberculosis*, *Shigella sonnei*, *Treponema pallidum*, and *Yersinia pestis* (genome sequences were obtained from http://bacteria.ensembl.org/index.html). The results shown in Table 5 indicate that in all the analyzed bacterial genomes, 1–2 repeat families could be detected at a statistically significant level. The least number of repeats were identified in the genome of *T. pallidum*, which may be due to its small size.

## 3. Discussion

In this study, we developed a new IP method and applied it to search for the families of dispersed repeats in the *E. coli* genome. As a result, we could identify three respective families of approximately 1.09 × 10^6^, 0.64 × 10^6^, and 0.58 × 10^6^ DNA bases (2.3 × 10^6^ bases in total), constituting almost 50% of the complete *E. coli* genome. Such extensive repeat families could not be detected in the *E. coli* genome via the RED, RECON, or Repeat_masker programs, but could be detected via the IP method, which could find de novo repeat families with *x* ≤ 1.5, whereas all other programs found them with *x* ≤ 1.0.

It should be noted that in search of the genomes containing 5 × 10^5^–1.1 × 10^7^ DNA bases, the level of false positives in each family was 145 ± 35 repeats. Such level of noise is due to the fact that, at the initial step of the iterative procedure, the random matrix can always find weak similarities (*Z* > 3.0) with some sequences (Figure 2). After creating a new PWM based on these similarities, the *Z* value for these sequences increases. In total, the iterative procedure can randomly include about 145 sequences in the PWM for which Z would be over 5.0.

A legitimate question arises regarding the origin of such highly divergent families of repeats and their functional significance. Our results (Section 2.4) indicate that each repeat has a similar triplet periodicity, which can account for the similarity of these sequences and classify them as one family. The emergence of triplet periodicity is partially related to the use of the same synonymous codons [31,32]. Therefore, the origin of repeat families could be associated with gene segments in which the same synonymous codons are used. In this case, dispersed repeats of the same family may be found in genes with similar transcriptional activity [33], whereas those with different activities could contain distinct families of dispersed repeats.

In Section 2.5 we found no correlation between the detected dispersed repeats and the binding sites of some of the proteins involved in nucleoid formation. Despite this, we cannot completely reject the assumption that the identified repeats contribute to DNA stacking in the nucleoid. Dispersed repeats create a certain markup of the bacterial genome that may contribute to the spatial self-organization of bacterial DNA.

Since dispersed repeats exist not only in the genome of *E. coli* but also in those of many other bacterial species (Table 5), it is also possible that the detected families of repeats could be involved in the creation of the liquid crystal structure within bacterial DNA through interactions between repeats within a family [34,35,36].

The IP method can be used to search for dispersed repeats in any DNA sequences, including those from eukaryotic organisms. The main limitation is that the analyzed sequence must be longer than 5 × 10^5^ bp. This limitation is due to the fact that the IP method uses an iterative procedure, meaning that at smaller lengths it is not possible to start because there will be no hits with Z > 3.0 (Figure 2). At the same time, for eukaryotic chromosomes with lengths more than 2 × 10^7^ bp the computation time could be too long. Therefore, the present version of IP can be used to find dispersed repeats in parts of eukaryotic chromosomes <2 × 10^7^ bp. The dispersed repeats found could then be used in nHMMER to search for IP-detected repeats in the whole genome.

The IP method is currently available on the server at: http://victoria.biengi.ac.ru/shddr, accessed on 29 June 2023, which is open for use. The search time for dispersed repeats in the *E. coli* genome was just over five days, and we plan to increase the capacity of this computational system as the number of users grows. If necessary, we will also increase the volume of the computer cluster and shorten the search time for dispersed repeats in a prokaryotic genome to about an hour or less.

## 4. Materials and Methods

Figure 2 illustrates the algorithm used in this work to search for dispersed repeats de novo. The algorithm is iterative and can be divided into six steps explained in detail below.

### 4.1. Calculation of the Random Matrix

To search for a family of dispersed repeats in sequence *S* with length *L*, we created a random PWM with 16 rows and *L*_1_ columns (step 1, Figure 2), in which *L*_1_ was the maximum repeat length that could be identified in sequence *S* using local alignment. The created matrix was then transformed so that sum $R^{2}=\sum_{i=1}^{16}\sum_{j=1}^{L_{1}}pwm{\left(i,j\right)}^{2}$ had constant value $R_{0}^{2}$ for all matrices used below to find the similarity between the PWM and sequence *S* (Section 4.2) (the procedure of matrix transformation is described in detail in [37]). For these matrices, sum $K=\sum_{i=1}^{16}\sum_{j=1}^{L_{1}}pwm(i,j)p_{1}(i)p_{2}(j)$ was also kept equal to *K*_0_. In this formula, *pwm*(*i*,*j*) is the element of the PWM on row *i* and column *j*, *p*_2_(*j*) = 1/*L*_1_ and *p*_1_(*i*) = *f*(*k*)*f*(*l*), where *f*(*k*) and *f*(*l*) are the probabilities of encountering nucleotides of types *k* and *l*, respectively, in the analyzed sequence *S* (Section 4.2) (*k* and *l* could be A, T, C, or G and pair *kl* formed index *i*).

In the present study, we used *L*_1_ = 600, *K*_0_ = −1.0, and $R_{0}^{2}=300L_{1}^{0.5}$; assuming that *K*_0_ = −1.0 permits the accurate determination of the start and end points of the local alignment [25] between the PWM and sequence *S*_1_ (Section 4.2). Thereafter, for local alignment we used the PWM with only these parameters.

### 4.2. Calculation of ${\bar{F}}_{\max}$ and σ for the PWM

To calculate ${\bar{F}}_{\max}$ and σ, we randomly shuffled sequence *S* (step 2, Figure 2). After choosing *t* = 1, in sequence *S* we selected a window (sequence *S*_1_) with the beginning at point *t* and end at point *t* + *L*_1_ + 50. If letter *N* occurs in sequence *S*_1_, then 10 should be added to *t* and sequence *S*_1_ should be created again.

Let *F_max_* be the maximum value of the similarity function after the local alignment between the PWM and sequence *S*_1_, performed by taking into account the correlation of neighboring bases in sequence *S*_1_ [38], and whose elements we have denoted as *s*_1_(*i*) for *i* from 1 to *L*_1_. Briefly, we first recoded the entire sequence *S*_1_, in which DNA bases became A = 1, T = 2, C = 3, and G = 4, and then created sequence *S*_2,_ in which elements *s*_2_(*j*) = *j* for *j* from 1 to *L*_1_ and which contained the column numbers of the PWM. Then, similarity function *F* was calculated as:

(2) $$\begin{array}{l} F(i,j)=\max\left\{\begin{array}{l} 0\\ F(i-1,j-1)+pwm(n,s_{2}(j))\\ F_{x}(i-1,j-1)+pwm(n,s_{2}(j))\\ F_{y}(i-1,j-1)+pwm(n,s_{2}(j)) \end{array}\right\} \end{array}$$

(3) $$\begin{array}{l} F_{x}(i,j)=\max\left\{\begin{array}{l} F(i-1,j)-d\\ F_{x}(i-1,j)-e \end{array}\right\} \end{array}$$

(4) $$\begin{array}{l} F_{y}(i,j)=\max\left\{\begin{array}{l} F(i,j-1)-d\\ F_{y}(i,j-1)-e \end{array}\right\} \end{array}$$

Initial conditions were: *F*(0,0) = *F*(*i*,0) = *F*(0,*i*) = 0.0 and *n* = *s*_1_(*k*) + 4(*s*_1_(*i*)−1)), where *I* and *j* each ranged from 2 to *L*_1_; for *i* = 1, *n* = *s*_1_(1) and for *j* = 1, *n* = *s*_1_(*i*). This choice for the initial *n* values had little effect on the final alignment results. By considering variable *n*, we took into account the correlation of neighboring nucleotides in sequence *S*_1_. To calculate *n*, we should find previous position *k*, which had already been included in the alignment and which had been calculated as previously described (Section 2.4 and Equation (7) in [38]). Here, we used *d* = 35.0 and *e* = 3.5.

First, we calculated matrix *F* and its maximum value *F_max_* for *t* = 1 and then added 10 bases to *t* and repeated the calculation up to *L*-*L*_1_-49. As a result, we obtained vector *Fmax*(*t*) and used it to calculate mean ${\bar{F}}_{\max}$ and σ for the used PWM. Together with matrix *F*, we filled in the matrix of inverse transitions, where each cell (*i*,*j*) had the coordinates of the cell or cells of matrix *F* from which we reached point (*i*,*j*). Then, we found coordinates (*i*_max_,*j_max_*) for *F_max_* and those for *F*(*i*_0_,*j*_0_) = 0 by backtracking. Thus, we obtained the local alignment of the PWM with sequence *S*_1_ and its coordinates (*i*_0_, *i_max_*).

### 4.3. Search for Similarities to the PWM in Sequence S

In sequence *S*, which was searched for dispersed repeats, we determined vector *F_max_*(*t*) (*t* = 1, 11, ..., *L*-*L*_1_-49) (step 3, Figure 2) using the PWM from Section 4.2. For each point *t* in sequence *S*, we calculated the coordinates of the beginning and end of local alignment *y*_0_(*t*) = *i_max_* and *y_max_* (*t*) = *i_max_* and then searched for local maxima in vector *F_max_*(*t*), which was found at position *t* if *F_max_*(*t* + *i*) < *F_max_*(*t*) for all *i* from *t*-*L*_1_-49 to *t* + *L*_1_ + 49. Next, we selected the local maxima for which *Z* = (*F_max_*−${\bar{F}}_{\max}$)**/**σ ≥ 5.0 and denoted the number of local maxima found in sequence *S* as *N_lm_*. For all found local maxima, the average *Z* was calculated as: $\bar{Z}=\sum_{k=1}^{k=N_{lm}}Z(k)/N_{lm}$.

Below, we show that the threshold value of *Z* > 5.0 provides about 6% of false positives for the first family of dispersed repeats found in the *E. coli* genome by this method, and that for the other two families the number of false positives was about 15%. As a result, we constructed local alignment of sequences *S*_1_ and *S*_2_ (columnar sequence of the PWM matrix) for each selected local maximum.

### 4.4. Creating a New PWM Based on the Found Similarities

Based on the obtained local maxima, we created a new PWM (Figure 2, step 4). For this, we used all local alignments found near the local maxima selected in Section 4.3, all of which had *Z* ≥ 5.0. These local alignments contained fragments of sequences *S*_1_ and *S*_2_ (Section 4.2); the former representing a nucleotide sequence in the numeric code and the latter representing PWM column numbers. Using sequences *S*_1_ and *S*_2_, we filled in frequency matrix *M*(16, 600) as *L*_1_ = 600 (Section 4.1). *M*(*n*,*s*_2_(*i*)) = *M*(*n*,*s*_2_(*i*)) + 1 for all *i* from 2 to *L*_1_ (*n* = *s*_1_(*i*−1) + 4*s*_1_(*i*)). Then, we calculated matrix of normal arguments *M*_1_(16,800) as:

(5) $$M_{1}\left(i,j\right)=\frac{M\left(i,j\right)-(L-1)p(i,j)}{\sqrt{\left(N-1)p(i,j)(1-p(i,j)\right)}}$$

where $p\left(i,j\right)=x\left(i\right)y\left(j\right)/{\left(L-1\right)}^{2}$, $x(i)=\sum_{j=1}^{L_{1}}M(i,j)$, $y(j)=\sum_{i=1}^{16}M(i,j)$, and $N=\sum_{i=1}^{16}\sum_{j=1}^{L_{1}}M(i,j)$. After the transformation of matrix *M*_1_ as described in Section 4.1, we obtained a PWM which could be used in Section 4.2.

### 4.5. Selection of the PWM to Find the Greatest Number of Similarities with Sequence S

To find a *PWM*(*j*) with the maximum value of $\bar{Z}$, the procedures described in Section 4.2, Section 4.3 and Section 4.4 were repeated *i* times (*i* = 1–20; step 5, Figure 2). The aim of these iterations was to find a *PWM*(*i*) with the maximum *i* value. The search was performed for *i* =1, 2, ..., 20, denoted as *i_max_*. As a result of iterations *i* = 1, 2, ..., 20, we memorized *PWM*(*j*) = *PWM*(*i_max_*), all alignments found for *i_max_*, their coordinates in sequences *S*_1_ and *S*_2_ (Section 4.3), and *Z* for each alignment. Then, the procedures described in Section 4.1, Section 4.2, Section 4.3, Section 4.4 and Section 4.5 were repeated *j* times.

### 4.6. Creating a Family of Dispersed Repeats

The procedures in Section 4.1, Section 4.2, Section 4.3, Section 4.4 and Section 4.5 were repeated 50 times, which means that index *j* varied from 1 to 50. Then, we chose the *j_max_* at which the maximum value (*i_max_*) was obtained (step 6, Figure 2) and obtained the first family of dispersed repeats. Thus, for each repeat family, we created *PWM*(*j_max_*) and all the alignments found for *j_max_*, obtained their coordinates in sequences *S*_1_ and *S*_2_ (Section 2.3), and determined *Z* for each alignment.

After creating the first family of repeats, we replaced the sequences of the found repeats in *S*_1_ with *N*, repeated the calculations described in Section 4.1, Section 4.2, Section 4.3, Section 4.4, Section 4.5 and Section 4.6, and constructed the next family of dispersed repeats.

## 5. Conclusions

We have developed a new mathematical method that allows identification of dispersed repeats with the average number of substitutions per nucleotide *x* ≤ 1.5, which is higher than that for any currently existing program. We have shown that all previously developed methods and algorithms (RED, RECON, and some others) can only find dispersed repeats for x ≤ 1.0. The new IP method has made it possible to detect families of dispersed repeats in bacterial genomes which have not been previously reported. We identify three families of approximately 1.09 × 10^6^, 0.64 × 10^6^, and 0.58 × 10^6^ DNA bases, respectively, constituting almost 50% of the complete *E. coli* genome. The length of the repeats is in the range of 400 to 600 bp. Other analyzed bacterial genomes contain one to three families of dispersed repeats with a total number of 10^3^ to 6 × 10^3^ copies. The existence of such highly divergent repeats could be associated with the presence of a single-type triplet periodicity in various genes or with the packing of bacterial DNA into a nucleoid. The method can also be applied for the search of dispersed repeats in eukaryotic genomes. We have created a web site for the analysis of bacterial genomes, where users can enter a genome sequence and obtain the result in a reasonable time.

## Figures and Tables

**Table 1:** Numbers of repeats identified in the four created repeat families using the IP method.

*x*	1	2	3	4
0	508	490	51	71
0.5	503	502	55	62
0.75	507	496	95	75
1.0	505	502	102	113
1.25	506	501	85	92
1.5	501	483	112	101
1.75	166	152	139	124
2.0	125	144	138	132
4.0	114	127	132	90

A model sequence of 4 × 10^6^ bases containing 500 repeats in the forward and 500 repeats in the reversed orientation was used; *x* is the average number of substitutions per nucleotide between family members.

**Table 2:** Numbers of dispersed repeats identified by the BLAST, nHMMER, and IP methods.

*x*	0.1	0.25	0.5	0.75	1.0	1.25	1.5	2.0	2.5	3.0	4.0	20.0
BLAST	1000	1000	1001	1000	1000	2.9	1.4	1.1	2.0	0.2	1.1	1.2
nHMMER	1004	1002	1004	1006	1002	1002	1004	1003	992	668	21	0
IP	1068	1006	1003	1002	1005	1002	1004	1003	1065	907	221	80

Model sequence *S*(*x*) of 4 × 10^6^ bases containing 10^3^ dispersed repeats was used; *x* is the average number of base substitutions per nucleotide between two dispersed repeats.

**Table 3:** Distribution of the found repeats from families 1, 2, and 3 (Figure 3) according to the proportion of non-coding regions.

Families	Proportion of Non-Coding Sequences									
	0.0–0.1	0.1–0.2	0.2–0.3	0.3–0.4	0.4–0.5	0.5–0.6	0.6–0.7	0.7–0.8	0.8–0.9	0.9–1.0
1	1956	129	58	36	22	7	3	2	1	25
2	918	97	60	32	13	11	4	4	3	28
3	709	116	72	43	29	15	8	2	3	27

**Table 4:** Matrices with dimensions 3 × 4 (A, B and C) which contain the normal distribution argument obtained by normal approximation of binomial distribution for each cell of matrices *M*_1_, *M*_2_ and *M*_3_.

DNA Bases	A			B			C		
	1	2	3	1	2	3	1	2	3
A	−48.7	−3.5	52.2	9.2	22.8	−32.0	24.7	3.9	−28.6
T	−9.8	−27.4	37.3	−40.4	7.7	32.7	−19.1	28.0	−8.8
C	17.2	37.1	−54.4	−20.4	−22.1	42.6	−6.8	−18.7	25.6
G	37.2	−8.6	−28.5	49.3	−7.13	−42.2	1.4	−12.1	10.7

Columns represent positions in a period of three bases; A, B, and C are matrices for sequences from the first, second, and third groups of dispersed repeats obtained for the escherichia_coli_str_k_12_substr_mg1655_gca_000005845 genome.

**Table 5:** Sizes of the first four groups dispersed repeats found in nine bacterial genomes.

Bacteria	1	2	3	4	Genome Size
*Azotobacter vinelandii*	4565	1357	322	178	5.3 × 10^6^
*Bacillus subtilis*	2563	768	340	305	4.2 × 10^6^
*Clostridium tetani*	1605	640	168	111	2.8 × 10^6^
*Methylococcus capsulatus*	2489	375	280	95	3.3 × 10^6^
*Mycobacterium tuberculosis*	3343	1152	299	103	4.4 × 10^6^
*Shigella sonnei*	2606	645	519	358	5.0 × 10^6^
*Treponema pallidum*	590	273	83	46	1.1 × 10^6^
*Xanthomonas campestris*	4622	1348	359	75	5.1 × 10^6^
*Yersinia pestis*	1953	43	35	43	4.8 × 10^6^

## Data Availability

The search for dispersed repeats in bacterial genomes can be performed on the website: http://victoria.biengi.ac.ru/shddr, accessed on 29 June 2023.

## Supplementary Materials

The supporting information can be downloaded at: https://www.mdpi.com/article/10.3390/ijms241310964/s1.
